# Development of an Individualized Prediction Calculator for the Benefit of Postoperative Radiotherapy in Patients with Surgically Resected De Novo Stage IV Breast Cancer

**DOI:** 10.3390/cancers12082103

**Published:** 2020-07-29

**Authors:** Byoung Hyuck Kim, Suzy Kim, Young Il Kim, Ji Hyun Chang, Ki-Tae Hwang, Sup Kim, Moon-June Cho, Jeanny Kwon

**Affiliations:** 1 Department of Radiation Oncology, Seoul Metropolitan Government Seoul National University Boramae Medical Center, Seoul 07061, Korea; karlly71@hanmail.net (B.H.K.); kimsuzy@hanmail.net (S.K.); 2 Department of Radiation Oncology, Chungnam National University School of Medicine, Daejeon 35015, Korea; minesota@cnuh.co.kr (Y.I.K.); supkim@cnuh.co.kr (S.K.); 3 Department of Radiation Oncology, Seoul National University Hospital, Seoul 03080, Korea; tweetiem@hanmail.net; 4 Department of Surgery, Seoul Metropolitan Government Seoul National University Boramae Medical Center, Seoul 07061, Korea; kiterius@gmail.com; 5 Cancer Research Institute, Chungnam National University Hospital, Daejeon 35015, Korea

**Keywords:** breast carcinoma, radiotherapy, nomogram, SEER

## Abstract

Purpose: Locoregional treatment has been increasingly adopted for metastatic breast cancer at presentation. This study aims to develop an individualized calculator to predict the benefit of postoperative radiotherapy (PORT) for patients with surgically resected de novo stage IV breast cancer. Methods and Materials: We searched the Surveillance, Epidemiology, and End Results (SEER) database for patients diagnosed with stage IV breast cancer between 2010 and 2014. After applying exclusion criteria, a total of 4473 patients were included in the analysis. Propensity score matching was used to balance the individual characteristics of the patients. After identifying the significant prognosticators, a nomogram was developed using multivariate regression models and internally validated. A web-based calculator was then constructed using a fitted survival prediction model. Results: With a median follow-up of 34 months, the three-year overall survival (OS) rates were 54.1% in the surgery alone group and 63.5% in the surgery + PORT group (*p* < 0.001). The survival benefit of PORT was maintained after propensity score matching (*p* < 0.001). Interaction testing of the prognostic variables found significant interactions between PORT and the presence of brain metastasis (*p* = 0.001), and between PORT and hormonal receptor expression (*p* = 0.018). After reviewing the performance of various models, a log-normal distributed survival model was adopted, with a C-index of 0.695. A calibration plot verified that the predicted survival rates were strongly correlated with the actual OS rates. A web-based survival calculator was constructed to provide individualized estimates of survival according to PORT. Conclusion: PORT significantly improved OS rates, though the individual benefit was affected by a number of factors. We successfully developed a nomogram and web-based calculator that predicted the prognosis according to PORT in patients with surgically resected de novo stage IV breast cancer. These tools are expected to be useful in clinical practice and in the design of related trials.

## 1. Introduction

Breast cancer is the most common tumors in women and is also a leading cause of cancer-related death worldwide [1]. About 5–10% of patients with breast cancer are diagnosed with de novo stage IV cancer [2]. The traditional treatment strategy for these patients is usually based on systemic therapy alone, such as chemotherapy (CTx), hormonal therapy, and/or targeted therapy, while surgery or radiotherapy (RT) are used to palliate the symptoms [3]. With advances in systemic therapy, the mortality rate for patients with stage IV breast cancer is decreasing but overall survival (OS) remains unsatisfactory [4]. This is partly due to the fact that stage IV breast cancer has a heterogeneous prognosis ranging from a few months to many years due to differences in tumor biology and metastatic disease burden [5,6,7,8].

In carefully selected patients, active locoregional treatment (LRT) has been attempted to increase survival outcomes. LRT can consist of surgery alone, RT alone, or both. Several retrospective series have reported an association between LRT and improved survival in patients with stage IV breast cancer at the time of diagnosis [9,10,11,12,13,14]. A meta-analysis including 28,693 patients found that surgical excision of the primary breast lesion improved the three-year survival from 22% to 40% (*p* < 0.01) [15]. Recently, the first randomized study (MF 07–01) comparing LRT followed by systemic therapy with systemic therapy alone reported a statistically significant improvement in median survival in the LRT group [16]. In that study, all patients who received breast-conserving surgery (BCS) received postoperative radiotherapy (PORT) to the whole breast as indicated in early breast cancer. However, recent phase III randomized trials investigating the role of LRT in a similar setting failed to prove that LRT was associated with higher survival rates [17,18]. Several ongoing trials investigating the same treatment approach are in the pipeline [19].

In addition to resections, additional PORT may also offer more robust locoregional control and greater potential for survival than surgery alone. Nevertheless, few clinical studies to date have evaluated the role of PORT in the treatment of surgically resected stage IV breast cancer. The decision to employ PORT is usually left to the judgment of the physician or to the multidisciplinary tumor board in most ongoing trials in the absence of specific guidelines.

Therefore, the current study aims to investigate whether PORT in addition to surgery improves survival outcomes compared to surgery alone in patients with de novo stage IV breast cancer, as well as to identify the factors which influence decision-making in terms of RT treatment for this group of patients. The results of this study will be provided to physicians in the form of a clinically available nomogram and a web-based calculator so that they can be helpful in practice.

## 2. Results

### 2.1. Patient Characteristics

The baseline characteristics of the 4473 patients included in the study are presented in Table 1. The median age of the cohort was 59 years (interquartile range (IQR), 49–68 years). About half of the patients had stage T3 or T4 cancer (47.1%) and N2 or N3 cancer (56.5%), indicating that the locoregional tumor burden was as significant as the metastatic tumor burden. The most frequently reported histologic grade was poorly differentiated (55.7%). Hormone receptor (HR) was positive in 74.6% and human epidermal growth factor receptor 2 (HER2) in 26.6% of the patients. In terms of distant metastases, bone metastasis was the most common (56.4%), followed by lung (23.2%), liver (19.3%), and brain metastasis (3.2%). BCS was conducted more frequently (71.7%) than a mastectomy (28.3%). CTx and RT were given to 67.7% and 40.0% of patients, respectively.

### 2.2. Survival Analysis for the Entire Cohort

**Figure 1:**
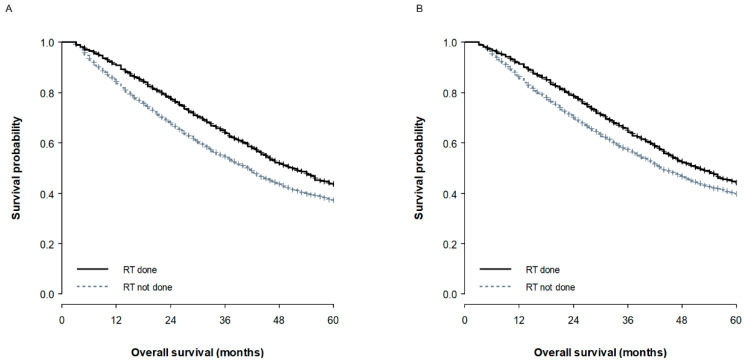
Overall survival curves according to the receipt of radiotherapy in the overall cohort (**A**) and the propensity score matched cohort (**B**).

The median follow-up duration was 34 months (IQR, 20–51 months). The results of the univariate and multivariate survival analyses for the entire cohort are summarized in Table S1. Most of the clinicopathological factors, including age, T or N stage, tumor grade, expression of HR or HER2, CTx, brain metastasis, and liver metastasis significantly affected OS, though type of breast surgery (mastectomy vs. BCS, *p* = 0.861) and the presence of bone metastasis (*p* = 0.984) did not. In particular, the three-year OS for the non-RT group was 54.1%, compared to 63.5% for the RT group (Figure 1A). Subsequent multivariate analysis produced similar results to the univariate analysis, though lung metastasis lost its prognostic significance (*p* = 0.577). RT remained statistically significant after adjusting for other prognostic factors (*p* < 0.001, hazard ratio 0.80; 95% confidence interval, 0.73–0.88).

### 2.3. Propensity Score Matched Survival Analysis

We compared the baseline characteristics between the non-RT and RT groups (Table 2). The patients in the RT group were younger (*p* < 0.001), had a more advanced T (*p* = 0.002) or N stage (*p* < 0.001), and were more likely to receive a mastectomy (*p* = 0.006) or CTx (*p* < 0.001) compared with the non-RT group. The RT group also included more patients with bone (*p* < 0.001) or brain metastases (*p* < 0.001), whereas lung metastases (*p* < 0.001) or liver metastases (*p* < 0.001) were more common in the untreated group. Propensity score (PS) matching was used to adjust for the observed imbalances; significant imbalances were not detected after this (Table 2, Figure S1).

The results of the survival analysis in the PS-matched cohort are presented in Table 3. Elderly (*p* < 0.001), black (*p* < 0.001), advanced T (*p* < 0.001) or N (*p* < 0.001) stage, high-grade tumors (*p* < 0.001), no expression of HR (*p* < 0.001) or HER2 (*p* < 0.001), no CTx (*p* < 0.001), non-RT (*p* < 0.001, Figure 1B), the presence of brain, liver, or lung metastasis (all, *p* < 0.001), and an increase in the number of metastatic sites (*p* < 0.001) were all significantly associated with a poor prognosis. Multivariate analysis was conducted on these variables using both semiparametric and parametric models. Of these models, the survival model using a log-normal distribution produced the lowest Akaike information criterion (AIC) (log-normal 17,281.7, Cox 25,605.3, Weibull 17,449.9, exponential 17,680.8, and log-logistic 17,293.7), which indicates the best performance. The log-normal survival model demonstrated that all of the included variables, except for the presence of lung metastasis, could independently affect OS.

**Figure 2:**
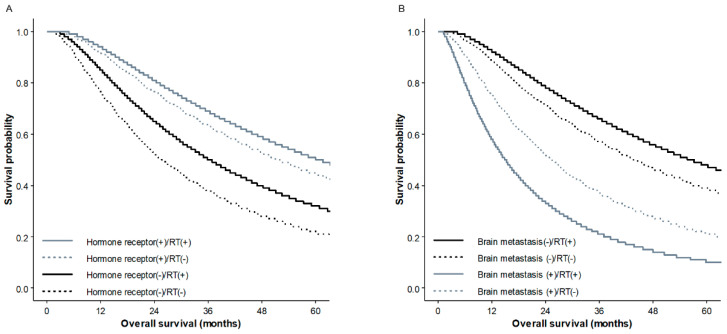
Overall survival curves according to hormone receptor status and/or radiotherapy (**A**) and the presence of brain metastasis and/or radiotherapy (**B**) in the propensity score matched cohort.

We also conducted interaction tests between the prognostic variables and found significant interactions between RT and brain metastasis (*p* < 0.001) and between RT and HR expression (*p* = 0.018). The benefit to survival of RT was higher in patients with HR (−) than in those with HR (+) (Figure 2A). In contrast, in patients with brain metastasis, the survival rate of those receiving RT was lower than those without RT, while the benefit to survival of RT was preserved in patients without brain metastasis (Figure 2B).

### 2.4. Development of a Nomogram and a Web-Based Survival Calculator

**Figure 3:**
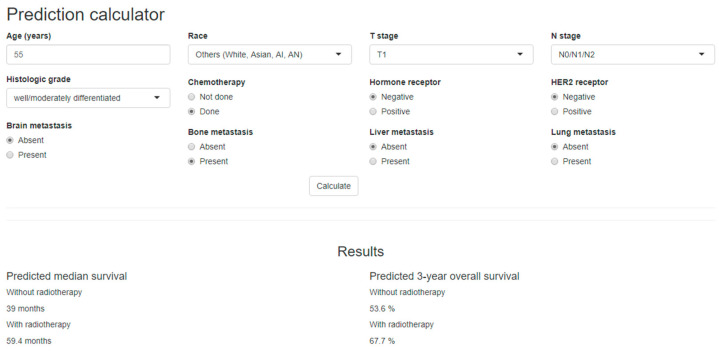
A web-based survival prediction calculator demonstrating the median survival and 3-year survival with or without radiotherapy in surgically resected stage IV breast cancer, which is available at http://bit.do/m1_nomogram.

Based on the results of the log-normal survival model, we developed a nomogram (Figure S2) and a web-based survival calculator (Figure 3) to predict the probability of survival in relation to PORT. The proposed nomogram was validated internally using 1000 bootstrap resamples, with a C-index of 0.695. A calibration plot verified that the predicted survival using our proposed nomogram was strongly correlated with actual OS (Figure S3). This web-based survival calculator is available from: http://bit.do/m1_nomogram. Using the survival calculator, we can assess the benefit of RT individually based on the entered clinical factors. For example, for a 50-year-old Caucasian patient with T2N2M1 breast cancer consisting of a moderately differentiated tumor and HR (−)/HER2 (−) accompanied by bone metastasis, the three-year survival rate is estimated to be 49.4% without RT and 63.9% with RT. In contrast, the three-year survival rate for a 50-year-old patient with T3N1M1 breast cancer consisting of a poorly differentiated tumor with HR (−)/HER (−) and brain, bone, and lung metastasis is expected as 12% without RT and 5% with RT.

## 3. Discussion

The use of LRT on the primary site in de novo stage IV cancer has recently received significant research attention, but its benefit still remains unclear for most forms of cancer. Accurately estimating the prognosis for an individual patient in this heterogeneous population can help clinical decision-making. We used the Surveillance, Epidemiology, and End Results (SEER) database to retrieve data from surgically treated stage IV breast cancer patients and developed a nomogram and a web-based calculator predicting the median survival and three-year OS for the use of PORT. As such, our results may help to improve patient selection for PORT, particularly as the use of this strategy becomes more widespread. Although the benefit to survival of PORT was demonstrated for both the overall and PS-matched cohort, this does not mean that it is beneficial to all patients. The potential use of PORT on patients that do not show a gain in OS using our proposed calculator should be carefully re-considered to avoid overtreatment with its associated increases in toxicities and cost.

Without an accurate method for predicting individual prognosis, relevant indication of LRT has not yet been established, and several conflicting results have been reported. In our interaction test, we observed an association between the benefit of PORT and both the type of metastasis and HR status. HR (−) patients exhibited greater improvement in survival following RT than did HR (+) patients. Interestingly, PORT had a harmful effect in patients with brain metastasis (Figure 2B). Similar findings have been observed in previous research, in which unplanned subgroup analysis of an MF 07-01 randomized trial found that the benefit of surgery was significant in patients with HR (+), HER2 (−), an age lower than 55, and solitary bone metastasis [16]. In addition, patients with liver/pulmonary metastasis had a worse prognosis with upfront surgery. Although the location of metastasis is not the same, these results suggested that some visceral metastasis may have predictive value for the benefit of LRT for stage IV breast cancer and may be used to select unsuitable candidates for primary site surgery with or without PORT. As expected, our results also demonstrated that patients with only one metastasis had a higher survival rate compared with patients with two or more metastases. Although many studies have demonstrated that RT on the primary site in patients with stage IV breast cancer is well tolerated, it is difficult to analyze the above problem in more depth due to various limitations in this study. One thing clear is that factors associated with OS, such as performance status, metastatic disease site/number, and the biologic tumor subtype which was associated with the use of hormone therapy or targeted therapy, should be all carefully considered for the successful employment of PORT in these patients [20].

Many previous studies have shown that a resection of the primary tumor in de novo stage IV cancer has a positive impact on survival, mostly after adjusting for other prognosticators [9,10,11,12,21]. However, most of these studies analyzed LRT in terms of surgery with or without RT or RT alone, whereas the role of PORT was not analyzed separately. For example, Choi et al. described a single institutional experience and reported significantly higher survival in the LRT group (five-year OS 73% vs. 45%, *p* = 0.02) [22]. They also found that surgery followed by RT had the most favorable outcomes (five-year OS 77% in surgery + RT, 70% in surgery alone, and 44% in RT alone) despite the small number of patients in each group [22]. Bourgier et al. also divided 308 metastatic breast cancer patients into two groups according to the type of LRT—RT alone or breast and axillary surgery with or without RT—but no difference in OS was observed between the two groups (hazard ratio for death 1.05; *p* = 0.83) [23]. However, it is difficult to draw definitive conclusions due to lack of research in this area. Despite this, it is worth noting that Hazard et al. reported that, for metastatic breast cancer patients, chest wall control was associated with better survival regardless of the type of primary surgical resection (hazard ratio 0.415; *p* < 0.0002) [24]. It is likely that this OS benefit could be supported by the addition of PORT after a resection. In a nomogram previously developed by other researchers to predict the OS of stage IV breast cancer using the National Cancer Database, RT was also independently associated with a decrease in the risk of death (hazard ratio 0.87, *p* = 0.007) [25]. Once the benefits of LRT have been more fully established, the role of detailed types of LRT (i.e., the extent of the primary resection, axillary dissection, and RT) will be further investigated.

There were several limitations in this study. In particular, its retrospective nature means that it might be biased towards patients with a better prognosis for the use of PORT; however, all of the selected patients had good performance status so that they were candidates for upfront surgery despite the presence of metastasis, and PS matching was also conducted, thus the impact of selection bias might not be significant. Another limitation was the lack of specific information concerning the use of endocrine therapy and anti-HER2 therapy, both of which have been consistently reported as independent prognostic factors for metastatic breast cancer. We adjusted all of the registered variables in the SEER database, but we were unable to consider other factors not collected in the database that may have affected the outcomes. Furthermore, because the SEER database provides only information on survival, we were unable to analyze the impact of LRT on locoregional control rate and therefore were unable to confirm whether the improvement of locoregional control led to an increase in survival. Lastly, specific information about PORT techniques (dose, fractionation, target volume, boost administration, and so on) and related toxicities was also not available. Recently, diverse radiotherapy techniques have been applied and radiation-induced toxicities are decreasing [26,27]. Nevertheless, potential risk and benefits should be considered together so that LRT in stage IV can be tailored to individual patients. Our results may help predict the ‘benefit’ aspect, but a prospective multicenter collaboration will still be needed to validate effectiveness of our developed tools.

## 4. Materials and Methods

### 4.1. Patient Cohort

We analyzed a cohort of 1,402,959 patients diagnosed with malignant breast cancer and registered with the SEER incidence data (1975–2016, Nov. 2018 submission) [28]. Of these patients, we identified 17,849 diagnosed with de novo stage IV breast cancer during the 2010–2014 period; however, given a wide scope of breast cancer histology, subjects for the study were limited to 14,604 patients diagnosed with the following histologic types using the approach of Li et al. [29]: ductal, lobular, ductal/lobular, mucinous, tubular, comedo carcinoma, inflammatory, medullary and papillary carcinoma. Of these patients, 4932 underwent surgery on the primary site, of which 4659 patients had RT information. Patients with bilateral breast cancer and those with occult breast cancer were excluded. While only those who received follow-up for more than three months were included because RT after surgery typically ends at least three months after diagnosis, leaving a final total of 4473 patients for analysis. This study followed the ethical standards of the institutional and/or national research committee (#07-2019-36) and with the 1964 Declaration of Helsinki and its later amendments or comparable ethical standards.

### 4.2. Clinicopathological Variables

We extracted information for the following clinicopathological variables from the patient data: age at diagnosis, race (white, black, American Indian/Alaska Native, Asian or Pacific Islander), sex, year of diagnosis, histologic ICD-0-3, grade, laterality, stage according to the American Joint Committee on Cancer (AJCC) Cancer Staging Manual 7th edition, extent of primary surgery, RT, CTx, hormone receptor (HR), HER2, initial distant metastasis (bone, brain, lung, or liver), and survival. Surgery was classified as either a mastectomy (code 20–24) or BCS (code 30–80). Radiotherapy was considered to have been done if the radiation sequence was “radiation after surgery” and considered to have not been done if the radiation sequence was “No radiation and/or cancer-directed surgery”. The number of metastatic sites was also investigated by summing the reports of distant metastasis (bone, brain, liver, and lung metastasis).

Several clinical variables (extent of primary surgery, HR, HER2, distant metastasis to specific site) were not available in some patients, but the percentages missing were small (the largest missing rate, 6.3% (284/4473 patients) of HR expression). RT and CTx were categorized as either “Done” or “Not done/unknown” according to SEER’s policy for completeness of variables. By SEER’s definition, “Not done/unknown” means that no evidence of RT/CTx was found in the medical records examined.

### 4.3. Statistical Analysis and the Development of a Nomogram and Web-Based Calculator

For the comparison between groups, χ^2^ tests and *t*-tests were used for the categorical and continuous variables, respectively. Since the patients treated with RT were not assigned randomly, PS matched analysis was conducted after employing the multiple imputation method for missing values. The imputation process was repeated until five different plausible datasets were obtained, which were then pooled to stabilize the results. Using the imputed dataset, the PS was calculated to predict the likelihood that RT was given to each patient. Based on the PS, patients were matched at a 1:1 ratio (RT group vs. non-RT group) using the nearest neighbor method. The distribution of the PS before and after PS matching is depicted in Figure S1.

OS was defined as the time from the date of diagnosis to the date of the last follow-up or death from any cause. The Kaplan–Meier method was used to estimate the survival curve, and log-rank tests were employed to compare the difference in survival rates for the categorical variables in univariate analysis. The factors proven to have a significant impact on survival were included in multivariate analysis using both semiparametric (the Cox proportional hazard regression model) and parametric models, including the Weibull, exponential, log-logistic, and log-normal regression models. The model with the lowest AIC was selected to develop a nomogram. In the parametric models such as log-normal regression, time ratios (TRs) were used instead of hazard ratios to describe the effects of individual predictors, indicating the acceleration factor for each variable for the time to an event [30]. Based on results of the multivariate analysis, we developed a nomogram to predict the survival benefit of the use of RT for individual patients. The developed nomogram was internally validated and calibrated using bootstrapping as assessed by the concordance index (C-index) and calibration curve. Using the obtained survival probability formula, we constructed a web-based survival calculator to simply compare the estimated outcomes with or without PORT. A *p*-value less than 0.05 was considered statistically significant, and all statistical analyses were performed using R version 3.5.1 (R Foundation for Statistical Computing, Vienna, Austria, http://www.r-project.org).

## 5. Conclusions

In conclusion, PORT significantly improved OS, though the extent of the benefit was affected by factors such as HR status and brain metastasis. Using the SEER database, we successfully developed a nomogram and web-based calculator predicting the individual survival benefit of RT in patients with surgically resected de novo stage IV breast cancer. These tools are expected to be useful in clinical practice and in the design of related trials.

## Figures and Tables

**Table 1:** Patient and Tumor Characteristics (*n* = 4473).

Characteristics		Number of Patients (%)
Age at diagnosis	Median (IQR), years	59 (49–68)
	≤50 years	1227 (27.4)
	51–70 years	2307 (51.6)
	>70 years	939 (21.0)
Sex	Male	62 (1.4)
	Female	4411 (98.6)
Race *	White	3380 (75.7)
	Black	732 (16.4)
	American Indian/Alaska Native	325 (7.3)
	Asian or Pacific Islander	27 (0.6)
Year of diagnosis	2010	1001 (22.4)
	2011	944 (21.1)
	2012	925 (20.7)
	2013	833 (18.6)
	2014	770 (17.2)
Laterality *	Right	2183 (48.9)
	Left	2285 (51.1)
T Stage *	1	653 (15.1)
	2	1638 (37.8)
	3	813 (18.8)
	4	1227 (28.3)
N stage *	0	292 (6.9)
	1	1532 (36.5)
	2	2343 (55.7)
	3	36 (0.9)
Histologic grade *	Well differentiated	292 (6.9)
	Moderately differentiated	1532 (36.5)
	Poorly differentiated	2343 (55.7)
	Undifferentiated	36 (0.9)
Hormone receptor *	Negative	1066 (25.4)
	Positive	3123 (74.6)
HER2 receptor *	Negative	3076 (73.4)
	Positive	1113 (26.6)
Bone metastasis *	Absent	1914 (43.6)
	Present	2479 (56.4)
Brain metastasis *	Absent	4203 (96.8)
	Present	137 (3.2)
Liver metastasis *	Absent	3525 (80.7)
	Present	842 (19.3)
Lung metastasis *	Absent	3347 (76.8)
	Present	1012 (23.2)
Breast operation	Mastectomy	1256 (28.3)
	Lumpectomy	3181 (71.7)
Chemotherapy	Not done/unknown	1447 (32.3)
	Done	3026 (67.7)
Radiotherapy	Not done/unknown	2683 (60.0)
	Done	1790 (40.0)

IQR, interquartile range. * Only cases with available information were analyzed.

**Table 2:** Comparison of Patient Characteristics According to The Use of radiotherapy before and after Propensity Score Matching.

		Unmatched Cohort			Propensity Score Matched Cohort		
Characteristics		Non-RT Group (*n* = 2683)	RT Group (*n* = 1790)	*p*	Non-RT Group (*n* = 1653)	RT Group (*n* = 1653)	*p*
Age at diagnosis	Median (IQR), years	61 (51–71)	57 (48–65)	<0.001	57 (48–66)	58 (48–65)	0.716
Race	White/others	2223 (82.9)	1517 (84.7)	0.094	1395 (84.4)	1401 (84.8)	0.773
	Black	460 (17.1)	273 (15.3)		258 (15.6)	252 (15.2)	
T stage	1	444 (16.5)	240 (13.4)	0.002	242 (14.6)	223 (13.5)	0.299
	2	1015 (37.8)	675 (37.7)		612 (37.0)	629 (38.1)	
	3	512 (19.1)	321 (17.9)		324 (19.6)	293 (17.7)	
	4	712 (26.5)	554 (30.9)		475 (28.7)	508 (30.7)	
N stage	0–2	2133 (79.5)	1331 (74.4)	< 0.001	1240 (75.0)	1239 (75.0)	0.968
	3	550 (20.5)	459 (25.6)		413 (25.0)	414 (25.0)	
Grade	1–2	1176 (43.8)	762 (42.6)	0.404	718 (43.4)	703 (42.5)	0.598
	3–4	1507 (56.2)	1028 (57.4)		935 (56.6)	950 (57.5)	
Hormone receptor	Negative	693 (25.8)	439 (24.5)	0.326	411 (24.9)	405 (24.5)	0.809
	Positive	1990 (74.2)	1351 (75.5)		1242 (75.1)	1248 (75.5)	
HER2 receptor	Negative	1981 (73.8)	1330 (74.3)	0.727	1230 (74.4)	1223 (74.0)	0.781
	Positive	702 (26.2)	460 (25.7)		423 (25.6)	430 (26.0)	
Breast operation	Mastectomy	721 (26.9)	549 (30.7)	0.006	493 (29.8)	487 (29.5)	0.819
	Lumpectomy	1962 (73.1)	1241 (69.3)		1160 (70.2)	1166 (70.5)	
Chemotherapy	Not done/unknown	1043 (38.9)	404 (22.6)	<0.001	412 (24.9)	395 (23.9)	0.491
	Done	1640 (61.1)	1386 (77.4)		1241 (75.1)	1258 (76.1)	
Bone metastasis	Absent	1230 (45.8)	726 (40.6)	<0.001	660 (39.9)	672 (40.7)	0.670
	Present	1453 (54.2)	1064 (59.4)		993 (60.1)	981 (59.3)	
Brain metastasis	Absent	2644 (98.5)	1681 (93.9)	<0.001	1614 (97.6)	1602 (96.9)	0.200
	Present	39 (1.5)	109 (6.1)		39 (2.4)	51 (3.1)	
Liver metastasis	Absent	2090 (77.9)	1522 (85.0)	<0.001	1381 (83.5)	1397 (84.5)	0.447
	Present	593 (22.1)	268 (15.0)		272 (16.5)	256 (15.5)	
Lung metastasis	Absent	1955 (72.9)	1483 (82.8)	<0.001	1370 (82.9)	1365 (82.6)	0.818
	Present	728 (27.1)	307 (17.2)		283 (17.1)	288 (17.4)	
No of metastatic site	0	483 (18.0)	399 (22.3)	<0.001	357 (21.6)	376 (22.7)	0.759
	1	1679 (62.6)	1101 (61.5)		1047 (63.3)	1025 (62.0)	
	2	432 (16.1)	229 (12.8)		210 (12.7)	417 (12.6)	
	3–4	89 (3.3)	61 (3.4)		39 (2.4)	84 (2.5)	

RT, radiotherapy; IQR, interquartile range; ALND, axillary lymph node dissection; ALNS, axillary lymph node sampling; LN, lymph node.

**Table 3:** Survival Analyses for The Propensity Score Matched Cohort.

Characteristics		3-Year OS (%)	Univariate Analysis ^a^	Multivariate Analysis ^b^	TR ^c^ (95% CI)
Age at diagnosis			<0.001	<0.001	0.99 (0.98–0.99)
Race	White/others	63.0	<0.001	<0.001	1
	Black	47.6			0.72 (0.65–0.80)
T stage	1	70.3	<0.001		1
	2	63.9		0.020	0.87 (0.77–0.98)
	3	59.9		0.001	0.80 (0.70–0.91)
	4	52.3		<0.001	0.71 (0.62–0.80)
N stage	0–2	62.3	<0.001	<0.001	1
	3	55.7			0.85 (0.78–0.92)
Grade	1–2	70.7	<0.001	<0.001	1
	3–4	53.1			0.74 (1.68–0.80)
HR	Negative	42.5	<0.001	<0.001	1
	Positive	66.6			1.93 (1.71–2.18)
HER2 receptor	Negative	56.4	<0.001	<0.001	1
	Positive	72.9			1.66 (1.52–1.82)
Chemotherapy	Not done/unknown	57.3	<0.001	<0.001	1
	Done	61.7			1.33 (1.21–1.47)
RT	Not done/unknown	57.1	<0.001	<0.001	1
	Done	64.1			1.47 (1.28–1.70)
Brain metastasis	Absent	61.5	<0.001	0.679	1
	Present	27.5			0.93 (0.67–1.30)
Liver metastasis	Absent	62.9	<0.001	<0.001	1
	Present	48.7			0.76 (0.67–0.87)
Lung metastasis	Absent	62.4	<0.001	0.331	1
	Present	51.9			1.06 (0.94–1.20)
No of metastatic site *	0	65.5	<0.001		1
	1	62.5		0.006	0.87 (0.79–0.96)
	2	49.1		<0.001	0.68 (0.57–0.81)
	3–4	29.9		<0.001	0.55 (0.40–0.75)
RT * Brain metastasis	RT done * Brain metastasis (−)			<0.001	1
	RT done * Brain metastasis (+)				0.41 (0.27–0.63)
RT * HR	RT done * HR (−)			0.018	1
	RT done * HR (+)				0.82 (0.70–0.97)

OS, overall survival; TR, time ratio; CI, confidence interval; HR, hormone receptor; HER2, human epidermal growth factor receptor 2; RT, radiotherapy. * No of metastatic site = bone metastasis + brain metastasis + liver metastasis + lung metastasis. ^a^ *p*-value by log-rank test. ^b^ *p*-value by log-normal multivariate regression model. ^c^ TR; the ratio denotes the acceleration factor on the time to an event (death). TR > 1 means that an event is less likely to occur. TR < 1 means that the event is more likely to happen.

## Supplementary Materials

The following are available online at https://www.mdpi.com/2072-6694/12/8/2103/s1, Figure S1: Distribution of propensity scores before and after matching; Figure S2: Nomogram used to predict median survival and 3-year overall survival. For each patient, we calculated the scores for the corresponding clinicopathological features and summed them to obtain the total. The predicted survival can be estimated based on the total score for each patient; Figure S3: Calibration plot suggested that the predicted 3-year survival agreed with the actual survival in the entire cohort; Table S1: Univariate and multivariate analyses for overall survival in the study population.
