# Supplementary material for: Development of an Individualized Prediction Calculator for the Benefit of Postoperative Radiotherapy in Patients with Surgically Resected De Novo Stage IV Breast Cancer
Source: Cancers (Basel). 2020 Jul 29;12(8):2103. doi: 10.3390/cancers12082103 (PMC7464221; doi:10.3390/cancers12082103)
Supplement: Supplementary file 1 [file cancers-12-02103-s001.pdf]

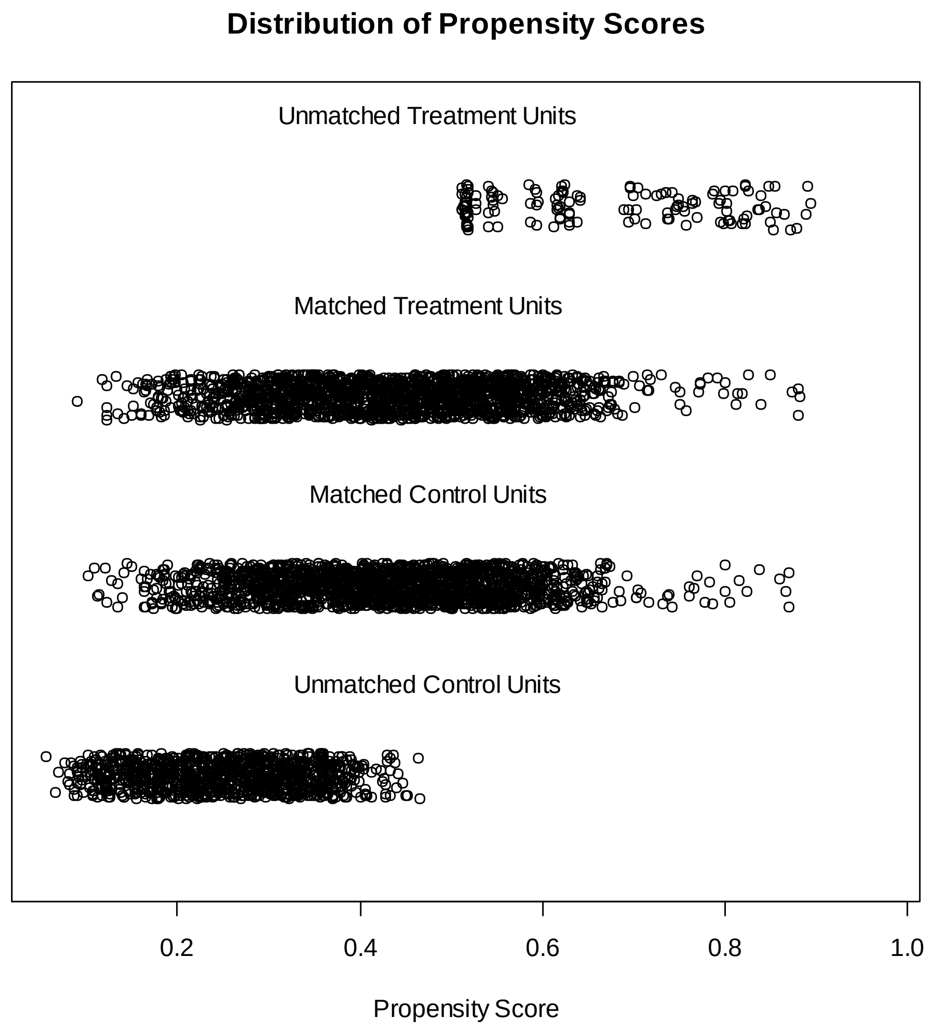
Distribution of Propensity Scores
Unmatched Treatment Units
Matched Treatment Units
Matched Control Units
Unmatched Control Units
0.2
0.4
0.6
0.8
1.0
Propensity Score

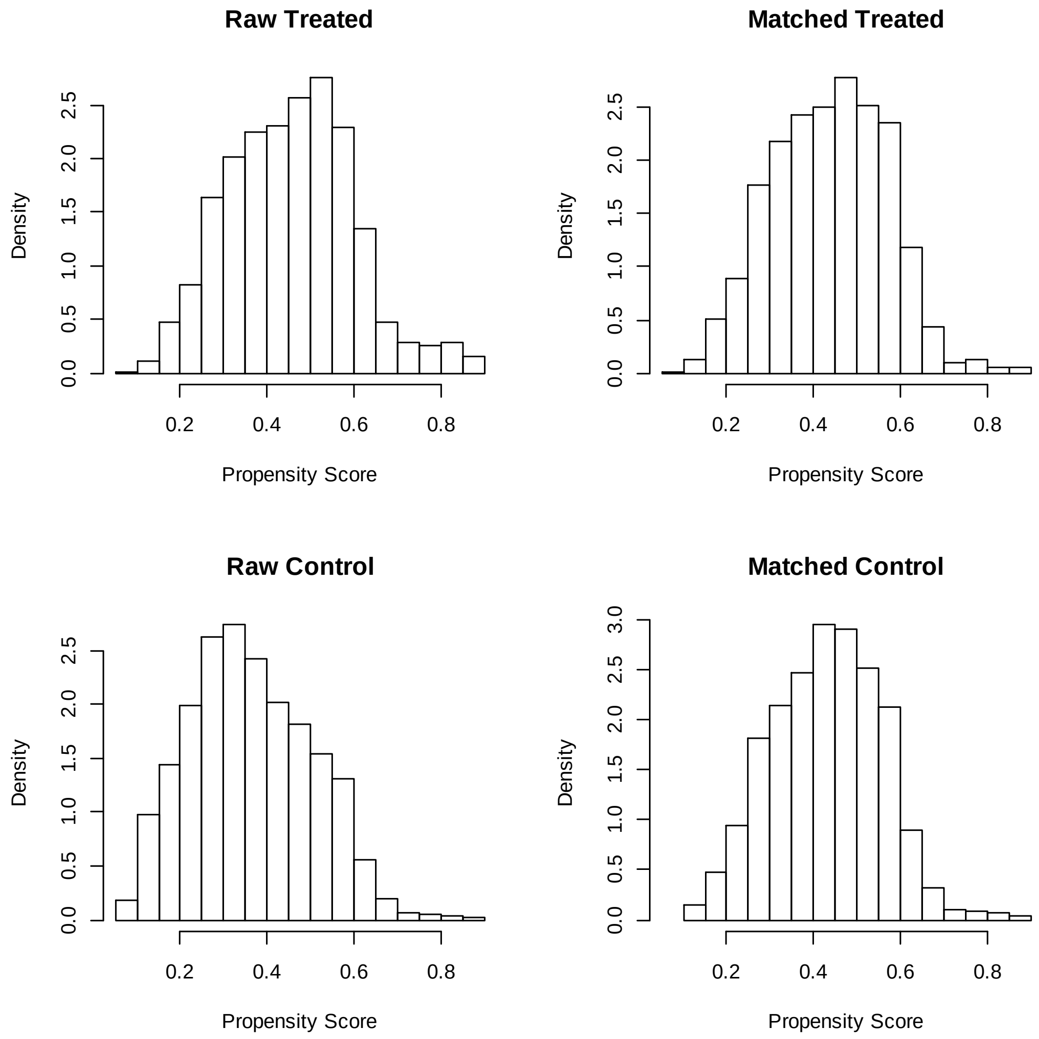
Raw Treated
Matched Treated
Raw Control
Matched Control
Density
Propensity Score
0.0
0.5
1.0
1.5
2.0
2.5
3.0
0.2
0.4
0.6
0.8

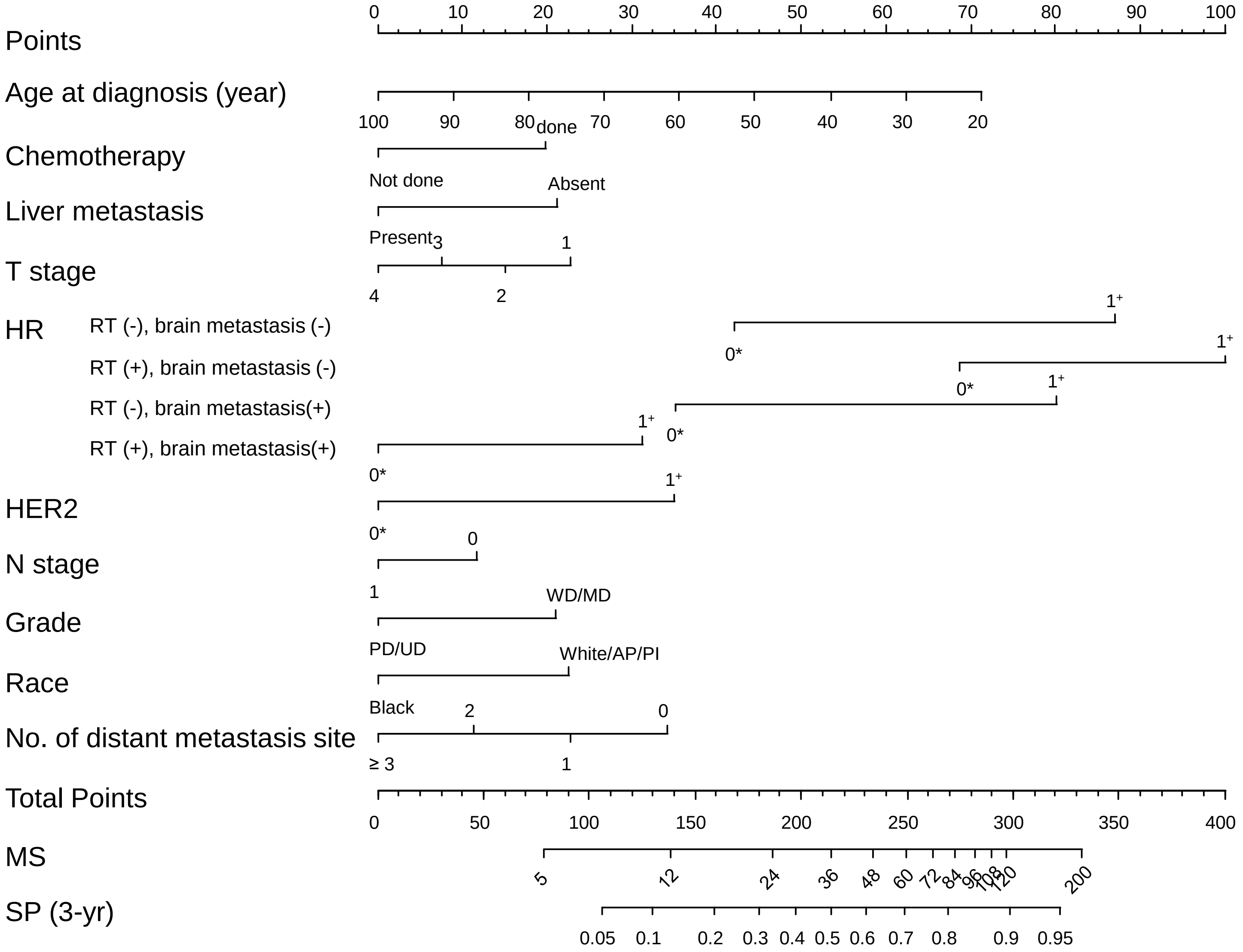
Points
0 10 20 30 40 50 60 70 80 90 100
Age at diagnosis (year)
100 90 80 70 60 50 40 30 20
Chemotherapy
done
Not done
Liver metastasis
Absent
Present
T stage
3 1
4 2
HR
RT (-), brain metastasis (-)
RT (+), brain metastasis (-)
RT (-), brain metastasis(+)
RT (+), brain metastasis(+)
1⁺
0*
HER2
1⁺
0*
N stage
0
1
Grade
WD/MD
PD/UD
Race
White/AP/PI
Black
No. of distant metastasis site
2 0
≥ 3 1
Total Points
0 50 100 150 200 250 300 350 400
MS
5 12 24 36 48 60 72 84 96 108 120 200
SP (3-yr)
0.05 0.1 0.2 0.3 0.4 0.5 0.6 0.7 0.8 0.9 0.95

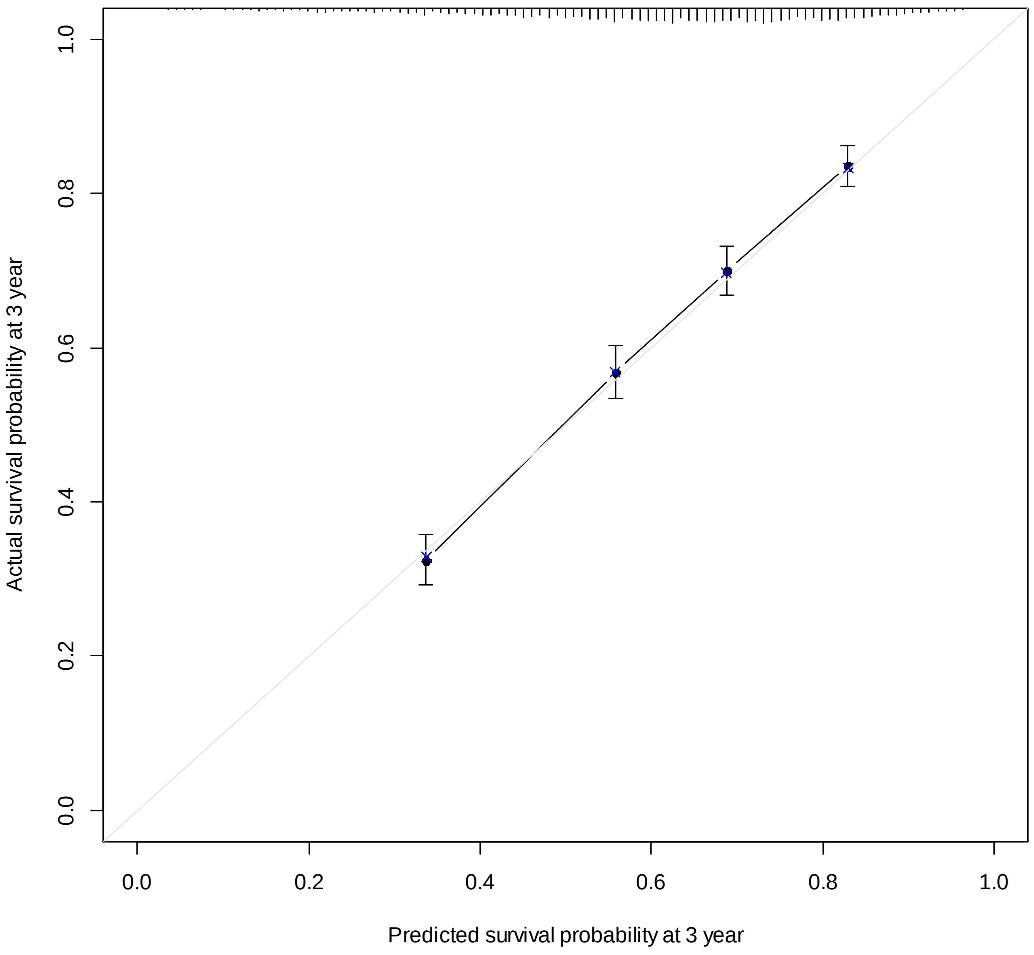

Actual survival probability at 3 year
Predicted survival probability at 3 year
0.0
0.2
0.4
0.6
0.8
1.0

**Supplementary Table 1. Univariate and multivariate analyses for overall survival in the study population**

| Characteristics | | 3-year OS (%) | Univariate analysis[a] | Multivariate analysis[b] | HR (95% CI) |
|---|---|---|---|---|---|
| Age at diagnosis | | | <0.001 | <0.001 | 1.02 (1.02-1.02) |
| Race | White/others | 60.4 | <0.001 | <0.001 | 1 |
| | Black | 45.1 | | | 1.35 (1.20-1.52) |
| T stage | 1 | 66.9 | <0.001 | | 1 |
| | 2 | 61.6 | | 0.008 | 1.23 (1.06-1.43) |
| | 3 | 56.9 | | <0.001 | 1.38 (1.16-1.63) |
| | 4 | 14.7 | | <0.001 | 1.60 (1.36-1.87) |
| N stage | 0-2 | 59.5 | <0.001 | <0.001 | 1 |
| | 3 | 54.4 | | | 1.29 (1.16-1.43) |
| Grade | 1-2 | 68.5 | <0.001 | <0.001 | 1 |
| | 3-4 | 49.8 | | | 1.46 (1.32-1.62) |
| Hormone receptor | Negative | 39.2 | <0.001 | <0.001 | 1 |
| | Positive | 65.0 | | | 0.50 (0.45-0.56) |
| HER2 receptor | Negative | 54.8 | <0.001 | <0.001 | 1 |
| | Positive | 68.5 | | | 0.48 (0.43-0.54) |
| Breast operation | Mastectomy | 58.3 | 0.861 | - | |
| | Lumpectomy | 57.9 | | | |
| Chemotherapy | Not done/uknown | 54.1 | <0.001 | <0.001 | 1 |
| | Done | 59.7 | | | 0.81 (0.72-0.90) |
| Radiotherapy | Not done/uknown | 54.1 | <0.001 | <0.001 | 1 |
| | Done | 63.5 | | | 0.80 (0.73-0.88) |
| Bone metastasis | Absent | 56.3 | 0.984 | - | |
| | Present | 59.6 | | | |
| Brain metastasis | Absent | 59.3 | <0.001 | <0.001 | 1 |
| | Present | 30.2 | | | 1.98 (1.55-2.52) |
| Liver metastasis | Absent | 61.2 | <0.001 | <0.001 | 1 |
| | Present | 46.0 | | | 1.49 (1.29-1.72) |
| Lung metastasis | Absent | 61.2 | <0.001 | 0.577 | 1 |
| | Present | 48.0 | | | 1.03 (0.90-1.18) |
| No of metastatic site* | 0 | 64.3 | <0.001 | | 1 |
| | 1 | 60.4 | | 0.012 | 1.18 (1.04-1.35) |
| | 2 | 48.1 | | <0.001 | 1.49 (1.21-1.82) |
| | 3-4 | 29.0 | | <0.001 | 1.82 (1.30-2.54) |

OS, overall survival; HR, hazard ratio; CI, confidence interval;

*No of metastatic site = bone metastasis + brain metastasis + liver metastasis + lung metastasis

[a]P-value by Log-rank test

[b]P-value by Cox proportional hazard model
